# The complete mitochondrial genome of the ‘solar-powered’ sea slug *Plakobranchus* cf. *ocellatus* (Heterobranchia: Panpulmonata: Sacoglossa)

**DOI:** 10.1080/23802359.2016.1247667

**Published:** 2017-02-22

**Authors:** Carola Greve, Maximiliano Ruiz-Tagle Lui, Sugirthan Sivalingam, Kerstin U. Ludwig, Heike Wägele, Alexander Donath

**Affiliations:** a Zoological Research Museum Alexander Koenig (ZFMK), Centre for Molecular Biodiversity Research (zmb), Bonn, Germany;; b Life & Brain Center, Department of Genomics, University of Bonn, Bonn, Germany;; c Institute of Human Genetics, University of Bonn, Bonn, Germany

**Keywords:** Gastropoda, kleptoplasty, mitochondrial, genome, mollusca, *Plakobranchus*, *cf. ocellatus*, sacoglossa

## Abstract

We present the complete mitochondrial genome sequence of *Plakobranchus* cf. *ocellatus* (Heterobranchia: Sacoglossa), a so-called ‘solar-powered’ sea slug with long-term retention of chloroplasts. The mitochondrial genome was 14,177 bp in length containing the standard set of 13 protein-coding genes, 2 rRNAs, and 22 tRNAs. The base composition of 27.3% A, 15.6% C, 18.6% G, and 38.5% T showed a strong A + T bias. The genome organization of *P.* cf. *ocellatus* is identical to the other sacoglossan mitogenomes sequenced so far, except for *Ascobulla fragilis*.

*Plakobranchus* cf. *ocellatus* van Hasselt, 1824 belongs to a small group of marine, heterobranch slugs, called Sacoglossa, of which certain species have the ability to sequester chloroplasts from its food algae (Hirose 2005; Christa et al. 2012). These ‘stolen’ plastids (kleptoplasts) are then stored in a functional state in the digestive gland cells of the slugs and allow them to endure weeks (short-term retention) or months (long-term retention) of starvation during which time the kleptoplasts continue photosynthesis inside the slugs (Trench et al. 1970; Christa et al. 2012, 2014; Wägele & Martin 2014; Vries et al. 2015). Here, we present the complete mitochondrial genome of *P.* cf. *ocellatus*, a so-called ‘solar-powered’ sea slug with long-term retention (Händeler et al. 2009; Christa et al. 2012).

Specimens of *P.* cf. *ocellatus* were collected on the Philippines (10°14'30.4"N 124°03'47.2"E) in December 2012. The heads of about 60 slugs were dissected to remove the post-pharyngeal nerve ring, which were directly deep frozen in liquid nitrogen and then stored (−80 °C). From these, genomic DNA was extracted using the QIAGEN DNeasy^®^ Blood & Tissue Kit (Hilden, Germany). Voucher material is stored in absolute ethanol at the Zoological Research Museum Alexander Koenig (voucher no. ZFMK-TIS-29490).

Two genomic libraries (insert sizes: 350 and 550 bp, resp.) were prepared using the TruSeq^®^ DNA PCR-Free Library Preparation Kit (San Diego, CA) according to the manufacturer’s protocol and 125 bp paired-end reads were sequenced on an Illumina HiSeq 2500 platform (San Diego, CA). All read pairs were used for mitochondrial genome assembly with MITObim 1.8 (Hahn et al. 2013), using the sequence of the mitochondrial cytochrome oxidase I gene of *P. ocellatus* (NCBI accession number: JX272720.1) as seed. After verification of correct circularity, genome annotation was done with MITOS revision 656 (Bernt et al. 2013), followed by manual correction using Geneious version 7.1.9 (Kearse et al. 2012).

The complete mitochondrial genome (GenBank accession number: KX853083) had a length of 14,177 bp encoding for 13 protein-coding genes, 2 rRNAs, and 22 tRNAs. The base composition is 27.3% A, 15.6% C, 18.6% G, and 38.5% T. A total of 183 bp nucleotides were observed in multiple small intergenic regions, ranging from 1 to 54 bp (found between the genes coding for COX3 and trnI(gat)). The gene order was as follows: trnK(ttt), cox1, trnV(tac), rrnL, trnL1(tag), trnA(tgc), trnP(tgg), nad6, nad5, nad1, trnW(tca), trnY(gta), nad4l, cob, trnD(gtc), trnF(gaa), cox2, trnG(tcc), trnH(gtg), -trnQ(ttg), -trnL2(taa), -atp8, -trnN(gtt), trnC(gca), -atp6, -trnR(tcg), -trnE(ttc), -rrnS, -trnM(cat), -nad3, -trnS2(tga), trnS1(gct), nad4, -trnT(tgt), -cox3, trnI(gat), nad2 and is therefore identical to the other sacoglossan mitogenomes sequenced so far, i.e. *Elysia chlorotica, E. ornata*, *Thuridilla gracilis*, and *Placida* sp. 1 NY-2013, except for *Ascobulla fragilis*.

The amino acid sequence of the 13 protein-coding genes of *P.* cf. *ocellatus* and all other sacoglossan species with fully sequenced mitogenome were extracted, aligned with MAFFT v7.271 (G-INS-i mode) (Katoh & Standley 2013), conspicuous sites as suggested by Aliscore v1.2 (Misof & Misof 2009) were removed, and remaining sites concatenated into a supermatrix. Optimal model parameters and the best partitioning scheme were searched with PartitionFinder v2.0.0-pre14 (Lanfear et al. 2014). Phylogenetic relationships, with partitioning and optimal model parameters being considered, were inferred (with 150 bootstrap replicates as found sufficient by the MRE-based bootstopping criterion) with RAxML version 8.2.9 (Stamatakis 2014) (Figure 1).

**Figure 1.**
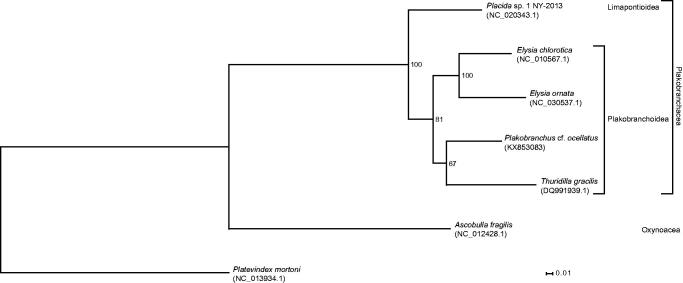
The molecular phylogeny of *Plakobranchus* cf. *ocellatus* and other sacoglossans (outgroup: *Platevindex mortoni*) based on the amino acid sequence of all protein-coding genes. The complete mitogenomes were downloaded from GenBank and the phylogenetic tree was constructed under the maximum-likelihood optimality criterion (150 bootstrap replicates).
